# Locating sex- and gender-specific data in health promotion research: evaluating the sensitivity and precision of published filters

**DOI:** 10.5195/jmla.2017.236

**Published:** 2017-07-01

**Authors:** Diane L. Lorenzetti, Yongtao Lin

## Abstract

**Objective:**

This study explored the effectiveness of search filters in identifying sex- and gender-specific data in health promotion studies that are indexed in MEDLINE.

**Methods:**

Literature searches were conducted to identify studies on patient or consumer attitudes and behaviors toward colorectal cancer screening, nutritional labeling, and influenza vaccination. Publications reporting sex- or gender-specific outcome data constituted the gold standards for this study. The sensitivity and precision of previously published gender-specific filters, as well as individual filter component terms, were calculated and compared with values identified in prior studies.

**Results:**

The sensitivity and precision of published sex or gender filters varied across topics. Sensitivity values ranged from 14.3% to 92.5%, while precision varied from 17.9% to 51.4%. These filters were less sensitive and less precise in their identification of relevant studies than has been reported in previous studies. Further, while the MEDLINE Medical Subject Headings (MeSH) term “Sex Factors” achieved the greatest average precision (59.3%) of any individual filter term, the MEDLINE check tag “Female” returned the highest average sensitivity (90.1%), with an average precision of 25.0% across topics.

**Conclusions:**

Although search filters can facilitate the identification of research evidence to enable decision making, variability in study abstracting and indexing can limit the generalizability and usability of these filters. This potential for variability should be considered when deciding to incorporate a search filter into any literature search. This research highlights the importance of this awareness when developing strategies for searching the published literature and the potential value of supplementing database searching with other methods of study identification.

## INTRODUCTION

Demographic and socioeconomic factors can affect individual decision making with respect to disease prevention and health promotion activities [1–4]. In recent years, researchers have increasingly emphasized the importance of tailoring health promotion interventions to specific populations [3]. Researchers have repeatedly cited the importance of careful consideration of the contexts in which health promotion interventions are situated and the populations that these initiatives are meant to benefit [1–4]. Sex (biology) and gender (socially constructed roles and behaviors) are key determinants of health that can impact individual “health status, health-seeking behavior and access to resources” [5]. As noted by Gelb and colleagues and Sparks, sex and gender influence individual decision making across a wide spectrum of life choices including, but not limited to, education, career, and health [6, 7]. As such, an awareness of the influence of sex and gender on health behaviors may be fundamental to increasing the reach and impact of health promotion initiatives [3].

Consideration of population-specific attitudes and preferences in the planning and implementation of health promotion initiatives can be informed through an understanding of relevant existing research on these issues. Given that there are currently more than 28,000 scholarly journals in publication, increasingly sophisticated strategies are required to enable researchers to identify literature that is most relevant to their specific needs [7, 8]. Search filters are combinations of keywords and subject headings designed to capture specific study designs, research methodologies, populations, geographic regions, or other themes of interest to searchers [9–14]. Combined with subject or topic searches, filters can enable the timely identification of research evidence relevant to specific lines of inquiry [11]. While a variety of search filters have been created to detect study designs such as randomized controlled trials and economic evaluation [9–14], recent efforts have also focused on identifying studies that report on age, race, and sex- or gender-specific outcomes [15–23].

In 2000, Montgomery and Sherif developed a MEDLINE search filter to retrieve sex- or gender-specific data for 6 areas that are relevant to the topic of women’s health (Table 1) [19]. The authors reported that this filter retrieved, “on average about 65% of the total pertinent articles reporting sex or gender differences” [19]. In 2009, Moerman and colleagues expanded on this research by developing 2 MEDLINE filters to enable the identification of clinical studies reporting outcome data for men and women [18]. One filter entirely comprised sex- and gender-relevant MEDLINE Medical Subject Headings (MeSH) terms, while the other combined keywords derived from an analysis of papers from a variety of clinical topics (Table 1). These filters, along with the previously published filter by Montgomery and Sherif, were tested against a set of Alzheimer’s disease and asthma studies published in core clinical journals [19]. Moerman and colleagues found that the Montgomery and Sherif filter returned a sensitivity/recall rate of 74% and a precision rate of 62%; the MeSH filter a sensitivity/recall rate of 31% and a precision rate of 79%; and their keywords filter a sensitivity/recall rate of 83% and a precision rate of 65% across these clinical areas [18]. In 2014, Stewart and colleagues created 2 highly sensitive filters to identify men’s health literature in MEDLINE and EMBASE (Table 1) [23]. The MEDLINE filter comprised keywords and check tags. Check tags are “concepts which are mentioned in almost every article (human, animal, male, female, child, etc)” [23]. These tags are “routinely added” to articles indexed in MEDLINE [23]. The Stewart filters were tested on subsets of literature on obesity management. The authors reported 100% filter sensitivity in MEDLINE; precision values, however, did not exceed 36% [23].

**Table 1:** Gender/sex-specific search filters (previously published)

Montgomery and Sherif [19]	Moerman Medical Subject Headings (MeSH) [18]	Moerman keywords [18]	Stewart A [23]	Stewart B [23]
1. Gender Identity/	1. Gender Identity/	1. (gender* or sex*).af	1. 1 not (women not men).tw	1. 1 and (male or males or men).tw
2. Sex Characteristics/	2. Sex Factors/	2. (boys or girls).tw	2. 1 not (female not male).tw	2. 1 and Male/
3. Sex Determination/	3. Sex Characteristics/	3. (women or men).ti	3. 1 and Male/	3. or/2–3
4. Sex Distribution/	4. Sex Distribution/	4. (male*1 or female*1).ti	4. or/1–3	
5. Sex Factors/	5. Sex/	5. (women or men).ab/freq=4		
6. Exp Women/	6. Sex Ratio/	6. (male*1 or female*1).ab/freq=4		
7. Women’s Health/	7. or/1–6	7. (women adj8 men).ab		
8. Women’s Health Services/		8. (female*1 adj8 male*1).ab		
9. Health Care		9. or/1–8		
For Women International.jn				
10. Journal of The American Medical Women’s Association.jn				
11. Women & Health.jn				
12. Womens Health Issues.jn				
13. female*.tw				
14. gender*.tw				
15. girl*.tw				
16. mother*.tw				
17. widow*.tw				
18. woman*.tw				
19. women*.tw				
20. or/1–19				

While the authors of these previous studies were able to demonstrate that sex- and gender-specific search filters can permit the identification of significant numbers of relevant studies, search filters do not always return similar results when tested against different study samples [14]. Search filter validation is an important element in the filter development process as it allows researchers to assess performance in the context of literature beyond what was used during initial filter derivation and testing [11]. The objective of this current study was to explore the effectiveness of previously published search filters in identifying sex- or gender-specific data in studies of health promotion interventions indexed in MEDLINE.

## METHODS

In the context of search filters, a gold standard is a predetermined sample of articles against which the performance of search filters is measured and established [11]. The authors chose three gender- and sex-neutral health promotion interventions from which to derive the gold standards for this study: colorectal cancer screening, nutritional labeling, and influenza vaccination. Our expertise informed the selection of the health promotion topics included in this study. Lorenzetti has coauthored systematic reviews on influenza vaccination uptake and nutrition labeling, and Lin has consulted on and participated in various studies focused on cancer screening and treatment [24–26].

## SEARCH STRATEGY

We performed comprehensive literature searches in Ovid MEDLINE (from inception to December 2010) to identify English language studies on patient or consumer attitudes toward colorectal cancer screening, nutritional labeling, and influenza vaccination. For all searches, intervention terms including colonoscopy, fecal occult blood, food labeling, nutrition information, and influenza vaccination were combined with patient or consumer attitude and behavior terms such as acceptance, attitude, behavior, satisfaction, and uptake to identify studies suitable for inclusion in the gold standard reference sets (supplemental appendix). Each term was searched as both title and abstract word and MEDLINE MeSH term, as appropriate. The abstracts of all studies retrieved from searching were downloaded into Reference Manager citation management software.

### Gold standard development and testing

All abstracts were independently screened by both authors for inclusion in the gold standard reference sets. Where abstract review was insufficient to establish study relevance, the full text was retrieved and reviewed by both authors. Disagreements (about 5% of study selection decisions) were resolved through reaching consensus. Original research studies were included if they focused on 1 of the 3 named health promotion interventions, targeted consumers or patients as the population of interest, and reported on gender- or sex-specific attitudes or behavior. The included papers constituted the gold standards for this study.

The five search filters published by Montgomery and Sherif, Moerman and colleagues, and Stewart and colleagues were applied (using the Boolean operator AND) to the topic searches outlined above (Table 1). These results were then compared to the reference sets to identify which gold standard studies could be identified by one or more filters. For all three health promotion topics, the performance of each filter was then calculated in terms of sensitivity (the total number of relevant papers retrieved as a proportion of the total number of relevant papers in the reference set) and precision (the total number of relevant papers as a proportion of the total number of papers retrieved by the filter). To assess the impact of publication date on our findings, we analyzed filter performance for both complete reference sets and subsets of reference articles published from 2005 to 2010. We also attempted to gauge the generalizability of each filter by computing average sensitivities and precisions across topics.

Collectively, the five search filters incorporate ten MeSH terms, eleven title or abstract words, and one check tag. To determine the performance of individual filter components, we calculated the sensitivity and precision of each MeSH term, check tag, and keyword found in one or more of the five filters, applying the same process outlined above. We also assessed the performance of one check tag (“Female”) and two MeSH terms (Men/and Men’s Health) not present in any filter, yet potentially relevant to gender or sex filter development.

## RESULTS

A total of 4,057 abstracts (1,980 colorectal cancer screening, 1,115 nutritional labeling, and 962 influenza vaccination) were identified from all 3 searches. Of these, 455 colorectal cancer screening, 106 nutritional labeling, and 195 influenza vaccination studies published from 1976 to 2010 were selected for inclusion and constituted the gold standard reference sets for this study.

The Montgomery and Sherif filter returned sensitivity values ranging from 33.8% to 62.0% and precision values from 19.9% to 51.3% for all studies included in our sample (Table 2). The Moerman MeSH filter produced sensitivity values from 6.7% to 17.0% and precision values of 30.5% to 64.7%. The Moerman keywords filter yielded sensitivity values ranging from 31.3% to 58.7% and precision values from 19.1% to 50.4% (Table 2). The Stewart A filter reported sensitivities between 88.1% and 99.5% and precision values from 9.3% to 21.8%; and the Stewart B filter generated sensitivities from 70.7% to 85.9% and precision values between 14.3% and 26.0% (Table 2). Sub-analyses by publication date did not reveal any consistent substantial differences in filter performance when reference sets were restricted to articles published in recent years (Table 2). While search filter performance varied across health promotion topics, filters typically returned higher precision values when applied to the colorectal cancer screening gold standard than the other two reference sets (Table 2).

**Table 2:** Search filter performance: all years (1976–2010) and most recent years (2005–2010)

	All gold standard papers (n)		All papers retrieved by filter (n)		Gold standard papers retrieved by filter (n)		Sensitivity (%)		Precision (%)	

Filter	1976–2010	(2005–2010)	1976–2010	(2005–2010)	1976–2010	(2005–2010)	1976–2010	(2005–2010)	1976–2010	(2005–2010)
Colorectal cancer screening										
Montgomery and Sherif (2000)	455	(263)	550	(314)	282	(165)	62.0%	(62.7%)	51.3%	(52.5%)
Moerman MeSH (2009)	455	(263)	119	(44)	77	(39)	16.9%	(16.7%)	64.7%	(88.6%)
Moerman keywords (2009)	455	(263)	530	(301)	267	(147)	58.7%	(55.9%)	50.4%	(48.8%)
Stewart A (2014)	455	(263)	1,838	(1,126)	401	(231)	88.1%	(87.8%)	21.8%	(20.5%)
Stewart B (2014)	455	(263)	1,502	(876)	391	(229)	85.9%	(87.1%)	26.0%	(26.1%)
Influenza vaccination										
Montgomery and Sherif (2000)	195	(135)	169	(110)	66	(49)	33.8%	(36.3%)	39.1%	(44.5%)
Moerman MeSH (2009)	195	(135)	32	(17)	13	(9)	6.7%	(6.7%)	40.6%	(52.9%)
Moerman keywords (2009)	195	(135)	146	(86)	61	(44)	31.3%	(32.6%)	41.8%	(51.2%)
Stewart A (2014)	195	(135)	960	(666)	194	(135)	99.5%	(100.0%)	20.2%	(20.3%)
Stewart B (2014)	195	(135)	639	(393)	159	(105)	81.5%	(77.8%)	24.9%	(26.7%)
Nutrition labeling										
Montgomery and Sherif (2000)	106	(54)	301	(143)	60	(29)	56.6%	(53.7%)	19.9%	(20.3%)
Moerman MeSH (2009)	106	(54)	59	(24)	18	(7)	17.0%	(13.0%)	30.5%	(29.2%)
Moerman keywords (2009)	106	(54)	241	(119)	46	(20)	43.4%	(37.0%)	19.1%	(16.8%)
Stewart A (2014)	106	(54)	1,111	(590)	104	(54)	98.1%	(100.0%)	9.3%	(9.2%)
Stewart B (2014)	106	(54)	524	(269)	75	(37)	70.7%	(68.5%)	14.3%	(13.8%)

The average sensitivity of filters across all 3 health promotion gold standards was calculated as: 54.0% (Montgomery and Sherif), 14.3% (Moerman MeSH), 49.5% (Moerman keywords), 92.5% (Stewart A), and 82.7% (Stewart B) (Table 3). The average precision of the published filters was found to be: 40.0% (Montgomery and Sherif), 51.4% (Moerman MeSH), 40.8% (Moerman keywords), 17.9% (Stewart A), and 23.4% (Stewart B) (Table 3). These averages are consistently less than values reported in previous studies.

**Table 3:** Search filter performance combined topics: all years (1976–2010) and most recent years (2005–2010)

	All gold standard papers (n)		All papers retrieved by filter (n)		Gold standard papers retrieved by filter (n)		Sensitivity (%)		Precision (%)	

Filter	1976–2010	(2005–2010)	1976–2010	(2005–2010)	1976–2010	(2005–2010)	1976–2010	(2005–2010)	1976–2010	(2005–2010)
Health promotion topics (combined)										
Montgomery and Sherif (2000)	756	(452)	1,020	(567)	408	(243)	54.0, 95% CI [50.5–58.0]	(54.0, 95% CI [49.0–58.4])	40.0, 95% CI [37.0–43.0]	(43.0, 95% CI [39.0–47.0])
Moerman MeSH (2009)	756	(452)	210	(85)	108	(55)	14.3 95% CI [11.9–17.0]	(12.1, 95% CI [9.4–15.6])	51.4, 95% CI [44.5–58.3]	(64.7, 95% CI [53.5–74.6])
Moerman keywords (2009)	756	(452)	917	(506)	374	(211)	49.5, 95% CI [45.9–53.1]	(47.0, 95% CI [42.0–51.4])	40.8, 95% CI [37.6–44.1]	(41.7, 95% CI [37.4–46.1])
Stewart A (2014)	756	(452)	3,909	(2,382)	699	(420)	92.5, 95% CI [90.1–94.2]	(93.0, 95% CI [90.0–95.0])	17.9, 95% CI [16.7–19.1]	(17.6, 95% CI [16.1–19.2])
Stewart B (2014)	756	(452)	2,665	(1,538)	625	(371)	82.7, 95% CI [79.7–85.3]	(82.1, 95% CI [78.2–85.4])	23.5, 95% CI [21.9–25.1]	(24.1, 95% CI [22.0–26.4])

An analysis of the component MeSH terms, title or abstract words, and check tags in the 5 filters and additional gender or sex MeSH terms (Men; Men’s Health) along with check tags (“Female”) excluded from these filters revealed that the check tags “Female” and “Male” returned the greatest overall average sensitivities (Female=90.1%; Male=79.2%) than any other term (Table 4). In contrast, check tags yielded relatively modest average precision values (Female=25.0%; Male=23.7%) across all reference sets (Table 4). As a counterpoint, while the MeSH term “Sex Factors” returned a relatively low average sensitivity (11.1%) across health promotion topics, it achieved the greatest average precision (59.3%) of any individual term (Table 4).

**Table 4:** Search filter performance: all years (1976–2010)

	Sensitivity (%)				Precision (%)			

	Colorectal screening	Nutrition labeling	Influenza vaccination	Average	Colorectal screening	Nutrition labeling	Influenza vaccination	Average
MeSH terms								
Gender Identity	0	0	0	0	0	0	0	0
exp Men	0	0	0	0	0	0	0	0
Men’s Health	0	0	0	0	0	0	0	0
Sex	0	0	0	0	0	0	0	0
Sex Characteristics	0	1.9	0.5	0.8	0	66.7	50.0	38.9
Sex Determination	0	0	0	0	0	0	0	0
Sex Distribution	2.9	1.9	0.5	1.8	38.2	20.0	12.5	23.6
Sex Factors	14.5	13.2	5.6	11.1	76.7	32.6	68.7	59.3
Sex Ratio	0	0	0	0	0	0	0	0
exp Women	0	0	0.5	0.2	0	0	100	33.3
Women’s Health	3.1	3.8	0	2.3	87.5	26.7	0	38.1
Women’s Health Services	0.2	0	0	0.1	100	0	0	33.3
Check tags								
Female	90.3	86.8	93.3	90.1	28.0	15.9	31.1	25.0
Male	84.0	68.9	84.6	79.2	26.4	15.1	29.6	23.7
Title/abstract words								
Boy(s)	0	0	0	0	0	0	0	0
Female(s)	15.4	16.0	8.2	13.2	64.8	21.0	40.0	41.9
Gender	18.2	7.5	11.8	12.5	61.0	17.0	54.8	44.3
Girl(s)	0	0	0	0	0	0	0	0
Male(s)	23.3	21.7	11.3	18.8	63.1	23.4	43.1	43.2
Men	33.0	10.4	3.1	15.5	55.0	18.6	21.4	31.7
Mother(s)	0.4	7.5	3.0	3.6	9.5	21.6	45.4	25.5
Sex	8.8	7.5	6.1	7.5	38.1	18.2	35.3	30.5
Widow(s)	0.2	0	0	0.1	100	0	0	33.3
Woman	0.9	0	0	0.3	44.4	0	0	14.8
Women	44.0	30.2	15.4	29.9	59.0	20.6	41.7	40.4

## DISCUSSION

Our study found that published sex- and gender-specific search filters were generally less sensitive and less precise in the identification of health promotion studies than was previously reported with clinically based reference sets [18, 23]. We also found little evidence to suggest that sex or gender filter performance improves when filter testing is restricted to literature published in the last ten years. In common with previous research on sex-specific filter development, MEDLINE check tags “Male” and “Female,” while highly sensitive, were less precise than any other term or term combination. While the Stewart filters yielded very high levels of sensitivity when applied to health promotion topics, both incorporated a check tag (“Male”) that resulted in low levels of precision relative to that of other filters in this study. The strength of our study is in the application of a systematic approach to exploring the performance of preexisting gender or sex filters in the context of health promotion research. Our study contributes to the literature on search filter development by highlighting the potential for variability in filter performance across topics.

A variety of factors can influence inconsistencies in search filter performance, both within and across individual studies. For instance, variations in precision can stem from discrepancies in the prevalence of gold standard studies (number of gold standard studies as a proportion of the total number of studies retrieved) identified in each topic area [27]. In our study, prevalence did vary across topics (nutrition labeling=9.5%; influenza vaccination=20.3%; colorectal cancer screening=23%). As such, one would expect the colorectal cancer screening set to yield higher prevalence values than other topics selected for this study.

While prevalence might have influenced the inconsistencies that we observed in this study, discrepancies in approaches to search filter testing can also affect filter performance. Gold standard reference sets commonly form the basis by which search filters are developed and tested [10]. Thus, variations in the methods by which these standards are derived, including the number and publication dates of studies in reference sets, can affect search filter performance across studies. For instance, Stewart and colleagues included 48 and Moerman and colleagues included 98 publications in their respective reference sets, whereas we incorporated a total of 756 gold standard studies (across all 3 health promotion topics) in this study [18, 23]. Similarly, while Moerman and colleagues included intervention studies in their gold standards, the reference sets for the current study included both intervention and observational studies [18]. These and other variations in filter design might have impacted the variability in filter performance that was observed in this study.

Further, while prior studies focused on filter development primarily in the context of clinically based topics, our study explored the effectiveness of filters in identifying outcome data in health promotion studies [18, 19, 23]. This could indicate more transparent reporting of sex-specific data in clinically based intervention studies. If true, this constitutes an important limitation regarding the general applicability of sex- and gender-specific filters across topics.

As has been noted in the literature, search filter performance fundamentally depends upon the standard application of relevant terms in both electronic database indexing (e.g., MEDLINE MeSH) and author-generated titles and abstracts [11]. When authors and indexers neglect to highlight the presence of sex-specific outcome data in titles, abstracts, indexing terms, and other searchable study fields, even highly sensitive search filters may be unable to identify relevant studies. Thus, the degree to which authors and indexers emphasize or de-emphasize elements of studies in these fields can impact the development and usability of such filters [11].

While filters that fail to identify any known studies are clearly flawed, no guidance currently exists for establishing the boundaries of high, medium, or low filter sensitivity or precision [28, 29]. In fact, Sampson and colleagues suggest that “in most information retrieval situations, a threshold will be explicitly or implicitly set for one parameter and efforts will be made to maximize the other” [30]. As noted by Jenkins, while some researchers wish to retrieve “all relevant literature,” others, including clinicians and health policy makers, may be willing to sacrifice a certain degree of sensitivity to ensure the precision, timeliness, and usability of the evidence that is retrieved [11]. Ultimately, it is essential that researchers, clinicians, and other decision makers are aware of the potential benefits and limitations of search filter usage. This awareness can enable searchers to make informed choices regarding filter adoption that reflect individual needs and circumstances.

This study has caveats and limitations. First, non-English language studies were excluded from our analysis. The inclusion of studies published in other languages might have yielded different findings. Also, because we were unable to determine the degree to which variations in filter sensitivity and precision impact the decision-making activities of researchers, health care professionals, or policy makers, the relative importance of the discrepancies in search filter performance reported in this study cannot be adequately assessed.

Ongoing validation of preexisting search filters with previously untested topics is an important means of establishing the boundaries of search filter performance. Information on the extent of search filter variability can assist researchers and health care professionals in making informed choices with respect to filter adoption. Future research efforts should continue to focus on validating sex-specific search filters against other reference standards and on exploring the degree to which search filter adoption impacts both literature search comprehensiveness and practice- and research-based decision making.

Although search filters can facilitate the identification of research evidence, variability in their performance may limit their generalizability and usability. This research highlights the importance of this awareness when developing strategies for searching the published literature.

## SUPPLEMENTAL FILE

Appendix Health promotion topics search strategies
