# Supplementary material for: Locating sex- and gender-specific data in health promotion research: evaluating the sensitivity and precision of published filters
Source: J Med Libr Assoc. 2017 Jul 1;105(3):216–25. doi: 10.5195/jmla.2017.236 (PMC5490698; doi:10.5195/jmla.2017.236)
Supplement: Appendix [file jmla-105-216-s.pdf]

JMLA Journal of the Medical Library Association

SC
Supplemental content to
J Med Libr Assoc. Jul;105(3): dx.doi.org/10.5195/jmla.2017.236
www.jmla.mlanet.org
© Lorenzetti, Lin 2017

Locating sex and gender-specific data in health promotion research: evaluating the sensitivity and precision of published filters

Diane L. Lorenzetti, PhD, MLS; Yongtao Lin, MLIS

APPENDIX

Health promotion topics search strategies

Colorectal cancer screening (Ovid MEDLINE)

1. colonoscopy/
2. colonoscop*.tw.
3. 1 or 2
4. exp colorectal neoplasms/
5. ((colorectal or colon or rectal) adj3 (neoplasm* or cancer*)).tw.
6. 4 or 5
7. mass screening/
8. early detection of cancer/
9. (routin* adj3 (test* or check* or diagnos* or detect*)).tw.
10. screening.tw.
11. ((faecal or fecal) adj3 occult adj3 blood).tw.
12. fobt.tw.
13. occult blood/
14. sigmoidoscopy/
15. sigmoidoscop*.tw.
16. colonography, computed tomographic/
17. (computed tomographic colonograph* or ct colonograph*).tw.
18. exp enema/ and exp barium sulfate/
19. barium enema*.tw.
20. dcbe.tw.
21. 7 or 8 or 9 or 10 or 11 or 12 or 13 or 14 or 15 or 16 or 17 or 18 or 19 or 20
22. 6 and 21
23. 3 or 22
24. attitude/ or attitude to health/ or health knowledge, attitudes, practice/ or behavior/
25. "patient acceptance of health care"/ or patient compliance/ or patient participation/ or treatment refusal/
26. health behavior/ or choice behavior/ or decision making/
27. consumer participation/ or consumer satisfaction
28. (accept* or attitude* or behavior* or behaviour* or choice or choose or choosing or decision making or practice or purchas* or select or selecting or uptake).tw
29. 24 or 25 or 26 or 27 or 28
30. 23 and 29
31. limit 30 to english language
32. limit 31 to (comment or editorial or letter)
33. 31 not 32
34. limit 33 to animals
35. limit 33 to (animals and humans)
36. 34 not 35
37. 33 not 36

JMLA Journal of the Medical Library Association

Supplemental content to
J Med Libr Assoc. Jul;105(3): dx.doi.org/10.5195/jmla.2017.236
www.jmla.mlanet.org
© Lorenzetti, Lin 2017

Nutrition labeling (Ovid MEDLINE)

1. food labeling/
2. ((food or nutrition* or diet*) adj10 label*).tw
3. (nutrition* adj5 information).tw.
4. 1 or 2 or 3
5. Limit 4 to english language
6. attitude/ or attitude to health/ or health knowledge, attitudes, practice/ or behavior/
7. "patient acceptance of health care"/ or patient compliance/ or patient participation/ or treatment refusal/
8. health behavior/ or choice behavior/ or decision making/
9. consumer participation/ or consumer satisfaction/
10. food habits/ or food preferences/ or feeding behavior/
11. (accept* or attitude* or behavior* or behaviour* or choice or choose or choosing or decision making or practice or purchas* or select or selecting or uptake).tw
12. 6 or 7 or 8 or 9 or 10 or 11
13. 5 and 12
14. limit 13 to (comment or editorial or letter)
15. 13 not 14
16. limit 15 to animals
17. limit 15 to (animals and humans)
18. 16 not 17
19. 15 not 18

Influenza vaccination (Ovid MEDLINE)

1. influenza vaccines/
2. (influenza adj5 (vaccin* or immuni*)).tw
3. 1 or 2
4. Limit 4 to english language
5. attitude/ or attitude to health/ or health knowledge, attitudes, practice/ or behavior/
6. "patient acceptance of health care"/ or patient compliance/ or patient participation/ or treatment refusal/
7. health behavior/ or choice behavior/ or decision making/
8. consumer participation/ or consumer satisfaction
9. (accept* or attitude* or behavior* or behaviour* or choice or choose or choosing or decision making or practice or purchas* or select or selecting or uptake).tw
10. 5 or 6 or 7 or 8 or 9
11. 4 and 10
12. limit 11 to (comment or editorial or letter)
13. 11 not 12
14. limit 13 to animals
15. limit 13 to (animals and humans)
16. 14 not 15
17. 13 not 16
